# Tumour-specific Causal Inference Discovers Distinct Disease Mechanisms Underlying Cancer Subtypes

**DOI:** 10.1038/s41598-019-48318-7

**Published:** 2019-09-13

**Authors:** Yifan Xue, Gregory Cooper, Chunhui Cai, Songjian Lu, Baoli Hu, Xiaojun Ma, Xinghua Lu

**Affiliations:** 1 Department of Biomedical Informatics, University of Pittsburgh School of Medicine, Pittsburgh, 15260 United States; 2 Department of Neurological Surgery, University of Pittsburgh School of Medicine, Pittsburgh, 15260 United States; 3 Paediatric Neurosurgery, UPMC Children’s Hospital of Pittsburgh, Pittsburgh, 15213 United States; 4 Molecular and Cellular Cancer Biology Program, UPMC Hillman Cancer Centre, Pittsburgh, 15232 United States

**Keywords:** Cancer models, Cancer genomics, Breast cancer

## Abstract

Cancer is a disease mainly caused by somatic genome alterations (SGAs) that perturb cellular signalling systems. Furthermore, the combination of pathway aberrations in a tumour defines its disease mechanism, and distinct disease mechanisms underlie the inter-tumour heterogeneity in terms of disease progression and responses to therapies. Discovering common disease mechanisms shared by tumours would provide guidance for precision oncology but remains a challenge. Here, we present a novel computational framework for revealing distinct combinations of aberrant signalling pathways in tumours. Specifically, we applied the tumour-specific causal inference algorithm (TCI) to identify causal relationships between SGAs and differentially expressed genes (DEGs) within tumours from the Cancer Genome Atlas (TCGA) study. Based on these causal inferences, we adopted a network-based method to identify modules of DEGs, such that the member DEGs within a module tend to be co-regulated by a common pathway. Using the expression status of genes in a module as a surrogate measure of the activation status of the corresponding pathways, we divided breast cancers (BRCAs) into five subgroups and glioblastoma multiformes (GBMs) into six subgroups with distinct combinations of pathway aberrations. The patient groups exhibited significantly different survival patterns, indicating that our approach can identify clinically relevant disease subtypes.

## Introduction

Cancer is a complex genetic disease, mainly caused by somatic genome alterations (SGAs) that affect oncogenic processes^1^. Such alterations include mutations, copy number alterations, DNA structure variants, epigenetic alterations, and other genomic variations^2^. Driver SGAs in a tumour activate the oncogenic process by perturbing genes in cellular signalling pathways that regulate homeostasis^2^. Cancers are heterogeneous in that tumours originating from the same tissue often exhibit significantly different molecular and clinical phenotypes, leading to differences in responses to treatments and patient survival. This well-known inter-tumour heterogeneity is largely due to distinct disease mechanisms underlying the development of an individual tumour, potentially resulting from different compositions of pathway aberrations. Understanding disease mechanisms of an individual tumour and further identifying common patterns of disease mechanisms among a cohort will not only provide insights into cancer biology but can also guide personalized therapy.

So far, it remains a challenge to discover disease mechanisms of cancers solely based on SGA data of tumours for the following reasons: First, a tumour usually hosts from hundreds to over a thousand SGA events^3^, among which the majority has relatively low-occurrence frequency in a tumour cohort. As a result. it is difficult to find sufficient patterns in SGA events. Second, among all the SGAs observed in a tumour, usually a small fraction directly promotes tumour development (drivers), whereas the majority of other SGAs is non-consequential with respect to oncogenesis (passengers)^2–10^. Identifying driver SGAs underlying the development of an individual tumour remains a major challenge in cancer genomics, which in turn makes it difficult to find co-occurrence patterns of cancer-driving SGAs. Third, an oncogenic pathway can be perturbed by different SGAs affecting distinct members of the pathway^9^. For example, the phosphoinositide 3-kinase (PI3K) pathway can be aberrantly activated by mutation/amplification of *PIK3CA*, mutation/deletion of *PTEN*, or mutation of *AKT1*^11,12^, and so on. There is no simple way to determine whether two distinct SGA events observed in two different tumours are affecting a common pathway. The above challenges make it difficult to use SGA data to determine which pathways are aberrant in a tumour and to further identify combination patterns of pathway aberrations.

On the other hand, gene expression profiles have been widely applied to identify molecular subtypes of cancers through clustering analysis, which leads to the discovery of subtypes among cancers originating from a common organ or tissue, and, in many cases, transcriptome-based subtyping reveals different outcomes and thus different responses to therapies^13–15^. However, while current approaches can identify genes differentially expressed in different subtypes of cancers, it is unclear which pathways drive their differential expression. Furthermore, current efforts in using gene expression patterns to find cancer subtypes can be heavily influenced by cell-type-specific expressions, leading to subtypes that are divided based on the origins of cells rather than disease mechanisms. For example, some breast cancer subtypes are based on the cell of origin, such as basal vs. luminal cells. In general, it would be ideal to identify a module of genes whose expressions are regulated by a specific oncogenic pathway, so that one can use expression status of such modules to discover combination patterns of pathway aberrations and classify tumours according to disease mechanisms rather than the tissue of origin.

**Figure 1:**
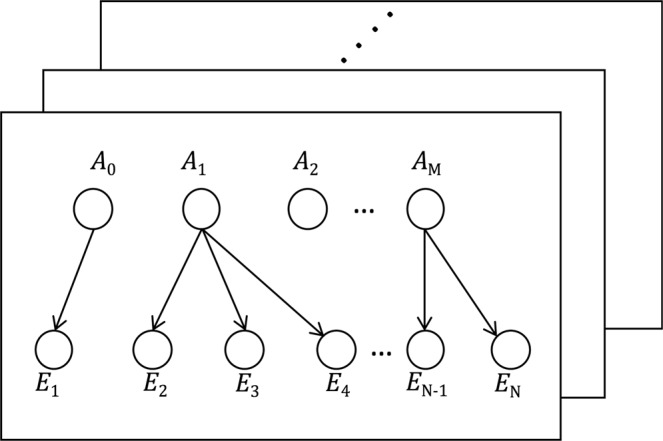
The diagram of the TCI algorithm. Each plate represents a tumour sample. Based on a causal Bayesian network model, TCI infers causal relationships between genes that carry somatic alterations (A) and genes that are differentially expressed (E). A_0_ designates all the factors other than gene alterations (e.g., the cellular environment). Each E receives one, and only one, A as its cause, and each A can be the parent of multiple Es.

In this paper, we present a novel end-to-end computational framework toward the goal of better understanding the disease mechanisms of each tumour. This framework transfers the information from genetic alterations to clinical outcomes via examining the expression modules that reflect the status of transcriptomic program perturbations. The framework is based on the results produced by a Bayesian causal learning algorithm we have developed and referred to as the Tumour-specific Causal Inference algorithm (TCI)^16^. TCI infers the causal relationship between SGAs and somatic genome alterations (DEGs) within an individual tumour (Fig. 1). This enables us to identify a set of DEGs that are causally regulated by a common SGA in a tumour as the signature of the pathway(s) perturbed by the SGA. Using TCI causal inferences, we adopt a network-based approach to construct a DEG network in which genes that are co-regulated by common SGAs are connected by weighted edges, and we apply spectral clustering on the network to identify modules of DEGs where the members share common driver SGAs. This enables us to use the expression status of a DEG module as a surrogate measure of the aberration status of pathways regulating its expression, which further allows us to represent a tumour as a vector in pathway space that reflects the combination of pathway aberrations in the tumour. With these pathway representative feature vectors, we identify subgroups of tumours sharing similar aberration patterns that exhibit different survival outcomes. We evaluated this computational framework on breast cancer (BRCA) and glioblastoma multiformes (GBM) data, and we report the results here. The same approach can be applied to other cancer types, with minor modification.

## Results

### DEG modules

We collected omics data of 5,097 tumours from the Cancer Genome Atlas (TCGA) dataset^17^. TCI analysis was applied to each of these tumours, which identified tumour-specific causal relationships between SGAs and DEGs in a tumour. Through a series of analyses of the pooled results from all tumours we further identified a set of candidate driver SGAs and their signature DEGs^16^. We then set out to construct a network of the signature DEGs of a specific cancer type to represent the co-regulation relationships among the DEGs. Specifically, each node in the network represents a DEG, and an edge was added between two DEGs if they were co-assigned to the same SGA by TCI in at least one-tenth of the tumours of this cancer type. The edge weight is proportional to the number of tumours in which the pair were co-regulated by a common driver SGA (note that the regulator SGA for a pair of DEGs can be different in different tumours; see Methods for more details). Our assumption is that the higher frequency that two DEGs are co-regulated by a common set of SGAs, the higher probability that these two DEGs are regulated by the same upstream signalling pathway perturbed by these SGAs. The DEG networks of BRCA and GBM were constructed using TCI results from 874 BRCA and 143 GBM tumours, respectively. The resulting networks contained 1,747 DEG nodes for BRCA and 3,576 DEG nodes for GBM.

**Figure 2:**
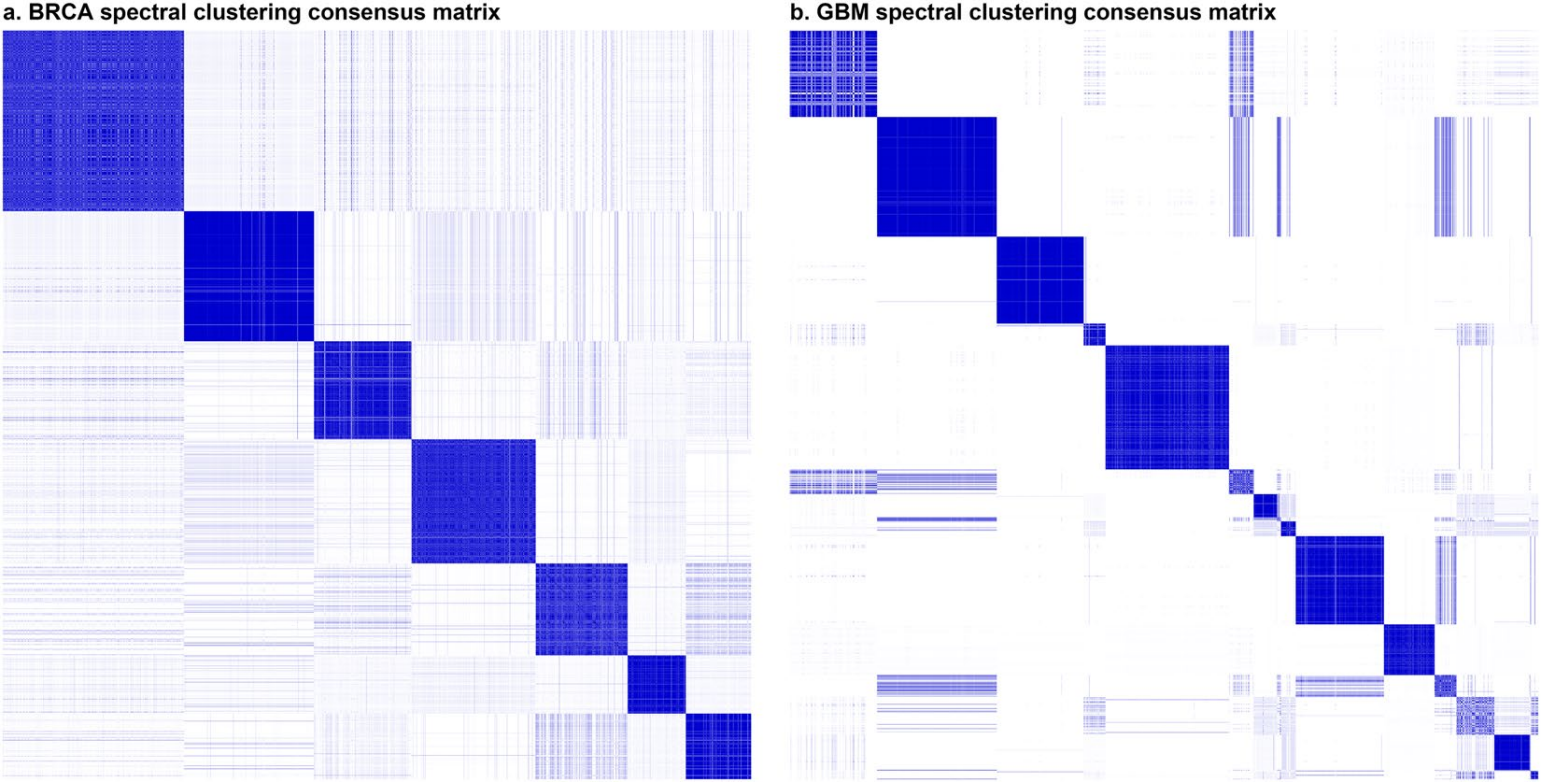
The consensus matrices of spectral clustering for identifying DEG modules. Spectral clustering was generated with 100 independent repeats of runs. The higher the frequency two DEGs were clustered into the same module, the darker blue the corresponding spot on the matrix. Each block sitting on the diagonal corresponds to a DEG module. The low overlapping across blocks indicates that spectral clustering was able to identify robust modules.

We then set out to identify modules of DEGs, such that each module consists of a set of DEGs that are likely co-regulated by a common pathway. The DEG modules were identified from the networks by implementing the spectral clustering algorithm^18^. Specifically, we repeatedly performed spectral clustering with different random initializations of cluster centres (see Methods) and then conducted a consensus clustering analysis by pooling the results and identifying DEGs that were consistently assigned to a common module during the experiment. Using this approach, we identified 7 DEG modules for BRCA and 15 for GBM, each containing from a few DEGs to over hundreds of DEGs (Fig. 2, Supplementary Tables S1 and S2). In comparison, when other more traditional clustering methods such as hierarchical clustering were used to search for DEG modules, the resulting DEG modules were inconsistent across independent runs with different random initializations (Supplementary Fig. S1). The results indicate that our approach can more reliably reveal DEG modules than other conventional clustering approaches.

To understand what function module each DEG module may represent, we ran a gene set overlap analysis on each DEG module against all gene sets in the Molecular Signature Database (MSigDB)^19^. The top 10 overlapping gene sets, according to the hypergeometric distribution p-value, are listed in Supplementary Tables S3 and S4. All BRCA DEG modules are correlated with some cancer-related gene sets, and most of them (modules 1, 3, 4, 5, 6 and 7) significantly overlap with breast cancer subtype-specific gene sets. For example, module 1 contains genes down-regulated in the luminal B subtype and genes up-regulated in the basal-like subtype. Similarly, half of GBM DEG modules overlap with tissue-specific gene sets, including those of neuron, synapse, and brain. Among the other modules, module 3 stands out with its enrichment of genes in MODULE_84, GO_IMMUNE_SYSTEM_PROCESS, and GO_IMMUNE_RESPONSE that represent immune and inflammatory responses. We hypothesize that module 3 represents a functional module that interacts with the immune system, which when it becomes defective helps a tumour escape immune surveillance. This conjecture, however, requires experimental study.

### Candidate pathways underlying DEG modules

Gene expression data have long been used to cluster tumour samples into subgroups, in which expression signatures associated with each subgroup are identified. However, it remains a challenge to determine which aberrant pathways drive the changed expression of signatures associated with such tumour subgroups and further utilize such information to understand distinct disease mechanisms. A key advantage of our framework is that we can estimate the SGAs underlying the co-regulation of members of a DEG module, i.e., the drivers of a DEG module that potentially perturb a common pathway. Each DEG module identified with spectral clustering contained a group of genes that were frequently co-regulated by the same set of SGAs. Accordingly, we extracted the SGAs that underlie the co-regulation for each DEG module. We called an SGA a dominant SGA of a DEG module if it produced over 10% of the co-regulation instances between DEG pairs in the module. Although for each DEG module there could have been hundreds of SGAs that contributed to its co-regulation instances, usually about three SGAs turned out to be dominant. The dominant SGAs together are responsible for about 90% or more of all the co-regulation instances. Different DEG modules had distinct dominant SGAs, although certain members overlapped (Tables 1 and 2). This indicates that each DEG module likely results from an upstream signalling pathway that is perturbed by a few major drivers.

**Table 1:** The composition of DEG modules of BRCA, including the number of DEGs, the number of effective DEGs, the dominant SGAs, and the proportion of co-regulations produced by each dominant SGA.

Module Index	# of DEG	# of Effective DEGs	Dominant SGAs (Prop. of Co-regulation)
Module 1	288	259	*CDH1* (60.2%), *GATA3* (20.8%), *PIK3CA* (12.4%)
Module 2	225	202	*PTEN* (66.1%), *PIK3CA* (19.6%)
Module 3	155	138	*ZFHX4* (44.7%), *RYR2* (22.9%)
Module 4	302	281	*GATA3* (92.7%)
Module 5	214	184	*ERBB2* (58.0%), *PIK3CA* (17.4%)
Module 6	135	124	*TP53* (96.4%)
Module 7	428	387	*PIK3CA* (90.5%)

**Table 2:** The composition of DEG modules of GBM, including the number of DEGs, the number of effective DEGs, the dominant SGAs, and the proportion of co-regulations produced by each dominant SGA.

Module Index	# of DEG	# of Effective DEGs	Dominant SGAs (Prop. of Co-regulation)
Module 1	413	255	*TP53* (99.69%)
Module 2	128	72	*PTEN* (50.0%), *SEC61G* (47.6%)
Module 3	529	347	*CDKN2A* (98.5%)
Module 4	170	81	*MARCH9* (97.9%)
Module 5	599	347	*PTEN* (98.3%)
Module 6	425	255	*SEC61G* (98.8%)
Module 7	11	7	*EGFR* (68.9%), *TP53* (31.0%)
Module 8	242	150	*CDKN2B-AS1* (94.8%)
Module 9	165	88	*AGAP2-AS1* (58.1%), *CHIC2* (41.4%)
Module 10	71	42	*CDKN2B* (94.2%)
Module 11	428	260	*EGFR* (97.9%)
Module 12	85	62	*CDKN2A* (69.2%), *PTEN* (30.1%)
Module 13	142	88	*GSX2* (75.0%), *RYR2* (11.4%)
Module 14	123	74	*MTAP* (94.5%)
Module 15	45	26	*TTN* (91.9%)

For BRCA, all dominant SGAs, except *ZFHX4* and *RYR2*, are well-known drivers of BRCA^13,20–22^. *ZFHX4* has been found to play a role in maintaining tumour cell state in GBM^23^, and our previous experimental study indicate that it does regulate the expression of certain target genes predicted by the TCI algorithm^16^. On the other hand, while some studies suggest that alterations on *RYR2* are likely passenger events, TCI consistently discovered that SGAs in RYR2 have impact on certain DEGs. Therefore, we propose *ZFHX2* and *RYR2* to be novel drivers for BRCA. For GBM, most dominant SGAs are known drivers of this cancer type^14,24,25^ except *MARCH9*, *AGAP2-AS1* (*AGAP2* antisense RNA 1), *CHIC2*, *GSX2*, *RYR2*, *MTAP*, and *TTN*. For these genes, except *MARCH9* and *TTN*, there is literature supporting that they are potential novel drivers of GBM. Specifically, *AGAP2-AS1* and *GSX2* are known to be associated with neuron system development^26,27^ and, therefore, alterations on these genes could be exclusive drivers of GBM. *CHIC2* has been found to be associated with myeloid leukemia^28^, and *MTAP* has been proposed as a tumour suppressor for BRCA^29^. For *MARCH9*, on the other hand, we consider it to be a passenger because it is on the same chromosome location 12q14.1 as *AGAP2-AS1*; they are frequently co-affected by the same genomic alteration event. *TTN* was found to be associated with BRCA and other cancer types^30,31^, but it is generally considered to be a passenger as its long polypeptide structure may bias its mutation frequency^15^.

Based on the dominant SGAs, we can infer what signalling pathway or function module each DEG module represents. *CDH1* and *GATA3* are the first two dominant SGAs of BRCA’s DEG module 1, and they are also two well-known drivers of BRCA^13,22^. 50.1% of TCGA BRCA samples (891 samples from the input data of TCI) have mutations in *CDH1*, *GATA3*, or *PIK3CA*, which suggests module 1 as the most associated function module with the disease mechanism of BRCA. With dominant SGAs *PTEN* and *PIK3CA*, DEG modules 2 and 7 represent the PI3K/Akt signalling pathway, which is known as one of the most commonly activated pathways in cancer^32^. The sharing of the dominant SGA *PIK3CA* across modules 1, 2, 5, and 7 suggests that although each module is considered to perform a relatively independent function, they are communicating with each other through interactions within a common signalling pathway. Module 3 contains two novel drivers, *ZFHX4* and *RYR2*, which cover 44.7% and 22.9% edges (pairs of DEGs) respectively. This may represent a novel functional module that would support the development of BRCA for some subgroups of patients (dominant SGAs mutations found in 18.2% samples). Module 4 has only one dominant SGA, *GATA3*, which represents the module resulting from a single driver rather than from the interactions between multiple drivers like module 1. Module 5, with its most dominant SGA being *ERBB2*, represents another important signalling pathway in BRCA, the ErbB/HER signalling pathway^33^. Module 6, on the other hand, represents the most commonly inactivated pathway in cancer, the p53 pathway^34^. Therefore, some of the DEG modules we identified for BRCA are more representative for general cancer signalling pathways, whereas others are more specific to a particular cancer type.

Similarly, in GBM, module 1 represents the p53 pathway. Modules 2, 5, and 12, sharing the dominant SGA *PTEN*, communicate with each other through the PI3K/Akt signalling pathway. Modules 3, 8, 10, and 12, with the most dominant SGA being *CDKN2s* (commonly deleted in GBM)^24^, represent function modules controlled by the cell cycle process. Modules 6, 7 and 11, with dominant SGAs being *SEC61G* and *EGFR* that were found specifically amplified in GBM^25,35^, represent the EGF/EGFR pathway. Modules 4, 9, 13, and 14, which have the most novel drivers, are potentially newly discovered functional modules that guide tumour development for some subgroups of GBM patients (dominant SGA mutations found in 19.7%, 28.9%, 24.6% and 39.4% samples, respectively).

### Identification of patient subgroups based on DEG module status

**Figure 3:**
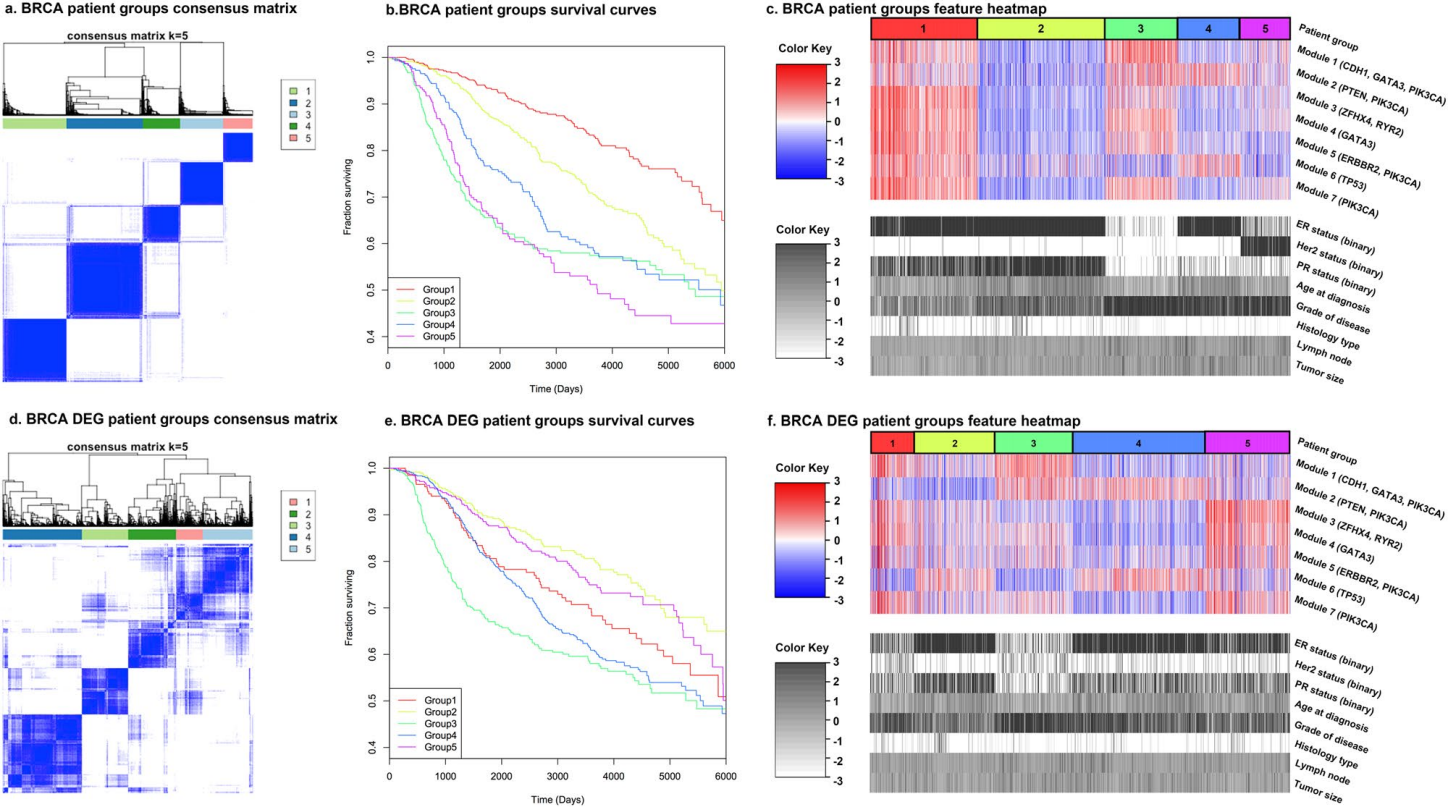
The consensus matrices of PAM consensus clustering for identifying patient groups for BRCA, the survival curves of the resulting patient groups, and the feature heatmaps. Patient groups were identified using all DEG modules and clinical features. DEG patient groups were identified using only DEG modules. For the heatmaps, the features were normalized across all patients. Values above 3 and below −3 are compressed into 3 and −3, respectively. The dominant SGAs of each DEG module are listed by the module index. The values of the clinical features of each DEG patient group are also given as a reference.

**Figure 4:**
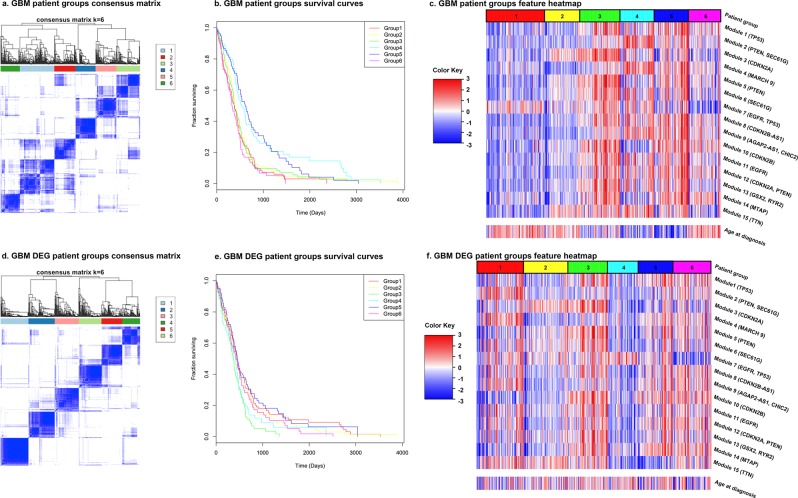
The consensus matrices of PAM consensus clustering for identifying patient groups for GBM, the survival curves of the resulting patient groups, and the feature heatmaps. Patient groups were identified using all DEG modules and clinical features. DEG patient groups were identified using only DEG modules. For the heatmaps, the features were normalized across all patients. Values above 3 and below -3 are compressed into 3 and -3, respectively. The dominant SGAs of each DEG module are listed by the module index. The values of the clinical features of each DEG patient group are also given as a reference.

Based on the hypothesis that the expression status of a DEG module would reflect the state of the pathway that regulates this module, we partitioned the BRCA and GBM patients into subgroups, using the expression status of the DEG modules as features (see Methods). To this end, we used the dataset from a study by the Molecular Taxonomy of Breast Cancer International Consortium (METABRIC)^20^, which has relatively complete gene expression and survival data of close to 2,000 breast cancer patients. For GBM, we used the gene expression and clinical data provided by the TCGA. The BRCA feature dataset used for clustering patients consists of the constructed DEG module features and 8 clinical features we had collected from the METABRIC dataset. The GBM feature dataset consists of the constructed DEG module features and age at diagnosis (the only clinical feature we considered, see Methods). Patient subgroups were identified using Partitioning Around Medoids (PAM, also known as k-medoids) consensus clustering, as consensus clustering generally produces more robust and consistent clusters^36^. PAM was selected, for it provides a centre of each resulting group with which new data can be classified, an advantage compared to the hierarchical clustering and it is generally more robust to noise and outliers than k-means^37^. When all clinical features and DEG modules were used, 5 and 6 patient groups were identified for BRCA and GBM, respectively (Figs 3a and 4a. Supplementary Tables S5 and S6). The Kaplan-Meier curves of patient groups (Figs 3b and 4b) show that different patient groups have different survival patterns. On average, BRCA patients have higher survival rates than GBM patients. This is consistent with the longer mean survival time of BRCA (2,951 days for our dataset) than GBM (510 days for our dataset). The p-value of the log-rank test for survival difference is <2 × 10^−16^ for BRCA and 8.96 × 10^−6^ for GBM, which suggests a significant difference between the survival distributions of the patient groups. For BRCA, group 1 has the best survival outcome, and group 5 has the worst survival outcome (Fig. 3b). For GBM, groups 4 and 5 have nearly twice the survival chance at the beginning compared to the other four groups (Fig. 4b).

Figures 3 and 4 also display the correlation between the features used in the PAM consensus clustering and the resulting patient groups as heatmaps. For BRCA (Fig. 3c), groups 1 and 2 have all clinical features alike and benign, which resulted in their significantly better survival outcomes compared to the other groups. The difference between their survival curves (Fig. 3b) is explained by their distinct patterns in DEG modules, with group 1 having significantly higher values than group 2. Group 3, the patient group with the second worst survival outcome (Fig. 3b), is a typical triple-negative group, with all three gene markers, estrogen receptors (ER), progesterone receptors (PR), and human epidermal growth factor receptor-2 (Her2) as negative. Group 4, with similarly lower DEG module values as group 2, distinguishes itself from group 2 with mainly PR negative patients and its high values in DEG module 2 (dominant SGAs *PTEN* and *PIK3CA*); its grade of disease is also higher, which resulted in its relatively lower survival chance. Group 5, having the worst survival outcome, contains most patients as Her2+. In summary, the survival of BRCA subgroups is strongly related to their clinical features such as age and protein-based biomarkers (ER, PR, and Her2). Given the similar clinical features, the pattern in DEG modules determines the survival difference. For GBM (Fig. 4c), groups 1 and 2 both contain older patients, which is associated with poor survival outcomes. Except that group 1 has specifically high value in module 7 (dominant SGAs *EGFR*, *TP53*) compared to group 2. Groups 3 and 4 distinguish themselves with their different distributions of DEG module values, especially in their reversed pattern in DEG modules 1–5. Although they both contain younger patients, their different values in DEG modules suggests that they have different combinations of signalling pathways being defective, which resulted in a much higher survival fraction of group 4 than group 3 (Fig. 4b). Group 5 contains most of the youngest patients, giving it the second-best survival outcome. Group 6, having the lowest average value in module 7, contains mostly older patients, making it indistinguishable from groups 1, 2, and 3 from a survival aspect. It can be seen that the age at diagnosis is the strongest indicator of survival chance of GBM, which agrees with previous studies that age has been found as strongly associated with GBM prognostic^38–43^. Given the similar patient ages, the pattern of DEG modules explains the difference between survival outcomes.

**Figure 5:**
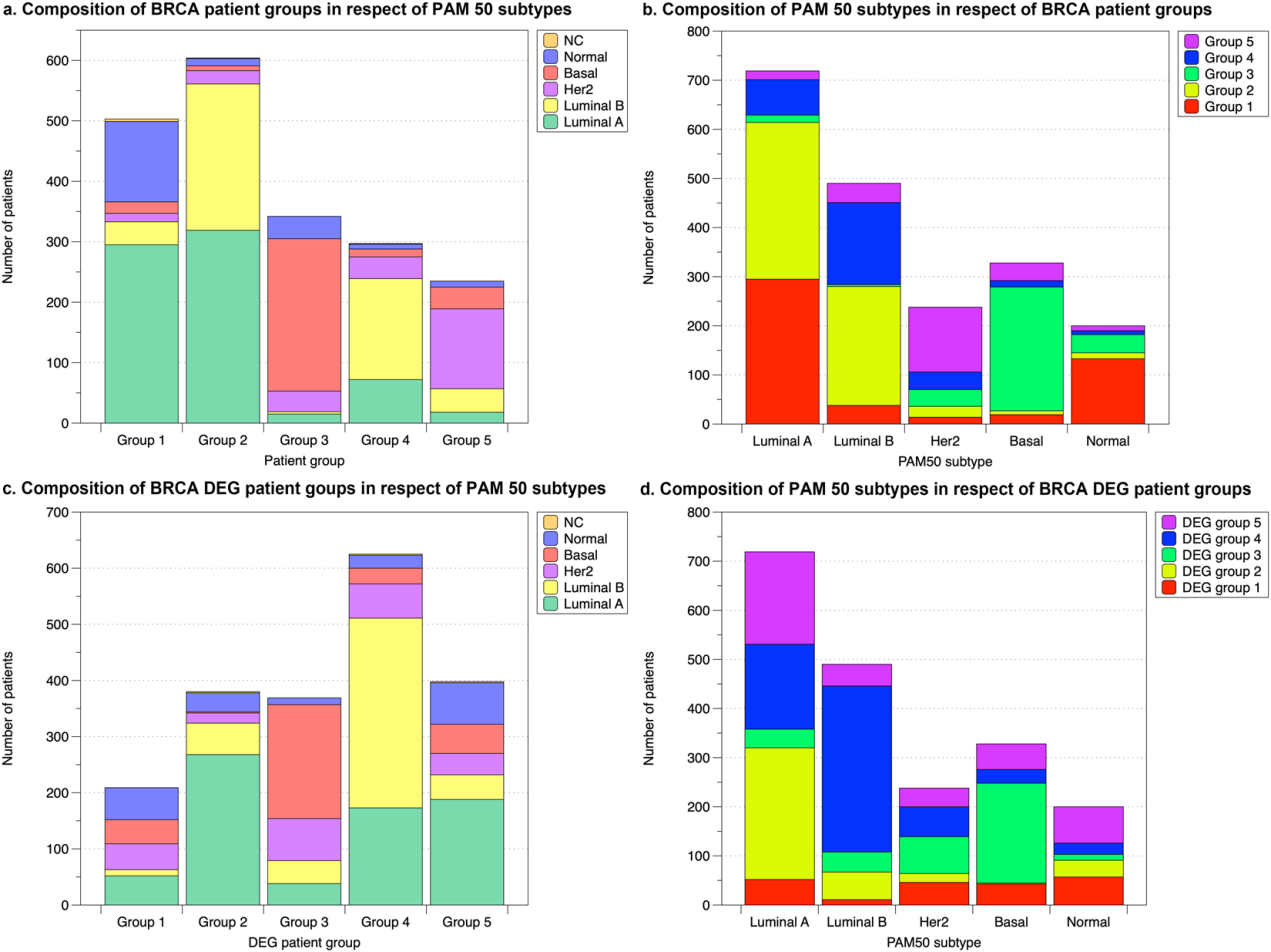
The comparison between BRCA patient groups and DEG patient groups with the PAM50 subtypes. (**a**,**c**) show the composition of patient groups/DEG patient groups in respect of PAM50 subtypes. (**b**,**d**) show the composition of PAM50 subtypes in respect of patient/DEG patient groups.

We next compared BRCA patient groups discovered by our approach with the PAM50 subtypes^13^ to see if these two patient classification standards correlate with each other (Fig. 5 and Supplementary Table S9). Each one of the five patient groups has a single dominant PAM50 subtype (overlapping proportion >50%). Groups 1 and 2 are mainly composed of luminal A patients (Fig. 5a). Specifically, luminal A and luminal B together make up over 90% of group 2. Group 4 is enriched in luminal B patients, followed by luminal A (Fig. 5a). Thus, groups 1, 2 and 4 together re-arrange the PAM50 luminal A and luminal B subtypes into three groups (Fig. 5b). The discovery of multiple subtypes in luminal/ER+ groups has been reported in previous studies^13,20^, which supports that a re-division of luminal subtypes is necessary. In addition, we also found that most ILC (invasive lobular carcinoma) patients and IDC (invasive ductal carcinoma) + ILC patients were clustered in patient groups 1, 2 and 4 (55.8%, 17.0% and 16.3%, respectively for ILC; 42.2%, 27.8%, and 17.8% for IDC + ILC). This agrees with previous studies that ILC patients are mostly ER + tumours classified as luminal A subtype^22^. Group 3, the triple-negative group, is dominated by basal-like patients (Fig. 5a), as basal-like tumours are typically negative for ER, PR, and Her2^13^. Group 5, the Her2+ group, is enriched in Her2 patients as expected (Fig. 5a). It is known that BRCA survival differs by subtype, and shortest survival is generally observed among Her2+ and basal-like subtypes^44^; this agrees with our observations of patient groups 3 and 5 on the Kaplan-Meier plot (Fig. 3b). There is no patient group that is mainly composed of normal-like patients. The p-value of survival difference between the PAM50 subtypes is <2 × 10^−16^. Therefore, both the PAM50 subtypes and our BRCA patient groups can efficiently divide BRCA patients into significantly different survival groups.

**Figure 6:**
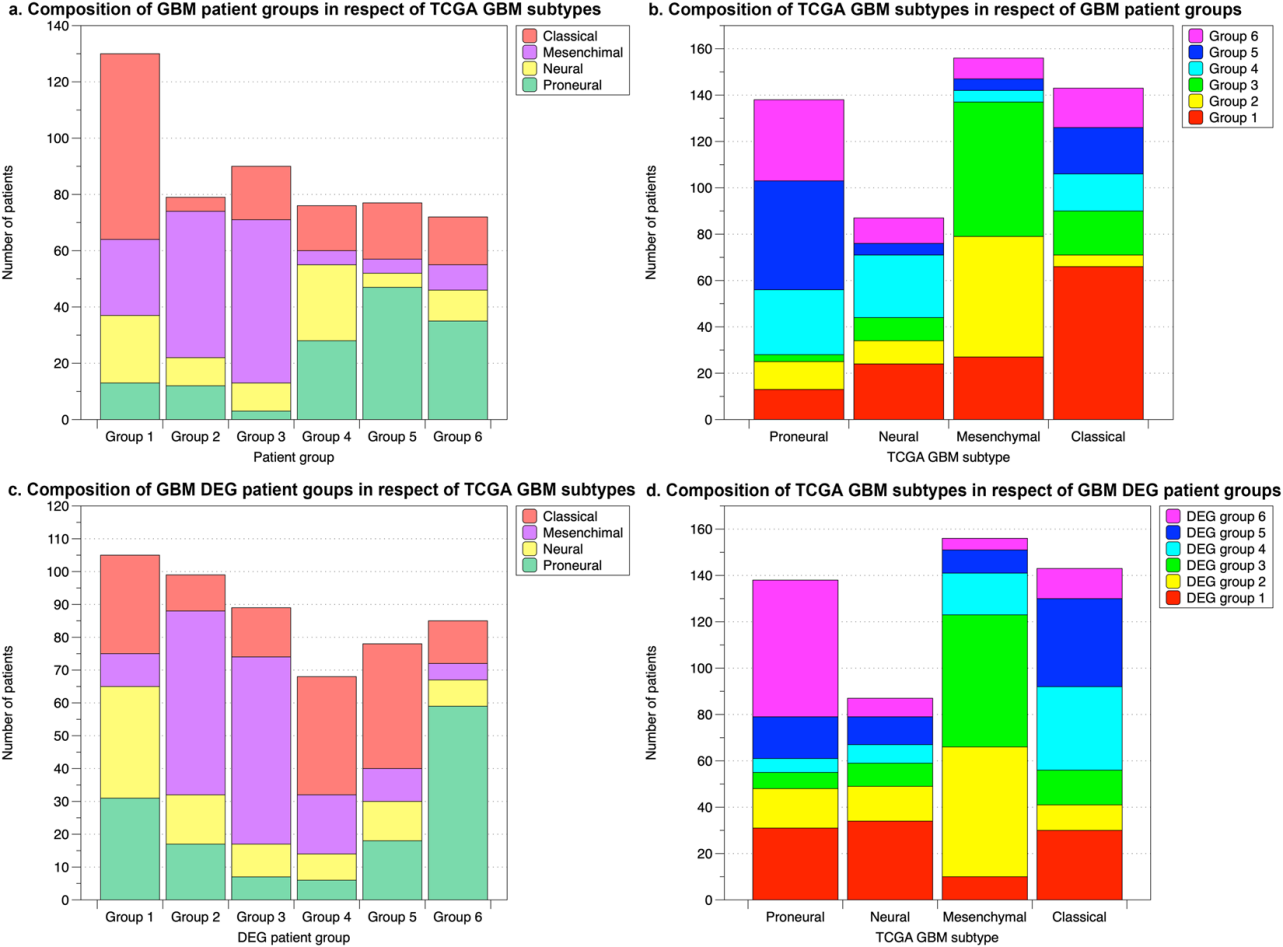
The comparison between GBM patient groups and DEG patient groups with the TCGA GBM subtypes. (**a**,**c**) show the composition of patient groups/DEG patient groups in respect of TCGA GBM subtypes. (**b**,**d**) show the composition of TCGA GBM subtypes in respect of patient/DEG patient groups.

We compared our GBM patient groups with the four GBM subtypes established by TCGA, 2010^14^ (Fig. 6 and Supplementary Table S10). Group 1 is mainly composed of Classical patients (Fig. 6a). Recall that group 1 has positive values in DEG module 7 (Fig. 4c), where the most dominant SGA is *EGFR*. *EGFR* was found to be highly amplified in the classical subtype, which supports the correlation between this subtype and patient group 1^14^. Groups 2 and 3 are both enriched in mesenchymal patients (Fig. 6a). These two groups consist of patients with different age ranges and DEG module distributions (Fig. 4c), which suggests intrinsic subgroups exist in mesenchymal patients. Group 5 is mainly composed of proneural patients, and nearly half of the patients in group 6 are also proneural (Fig. 6a). The neural subtype has been considered as normal tissue contamination, thus it is not an intrinsic subtype of GBM^45^. This is consistent with our observation that no patient group we identified is strongly enriched in neural patients. The p-value of the log-rank test of GBM TCGA subtypes is 0.06, significantly higher than that achieved by our GBM patient groups (8.96 × 10^−6^), which indicates that the GBM patient groups are more survival indicative compared to the TCGA subtypes.

To examine the power of genetic features alone in predicting patient survival outcome, a second PAM consensus clustering of patients was completed using only the DEG modules as features. This also gave rise to a division of BRCA data into 5 patient groups, and a division of GBM data into 6 patient groups (Figs 3d and 4d. Supplementary Tables S6 and S8). For simplicity, from now on we will refer to these patient groups as the DEG patient groups. Although the survival curves of these DEG patient groups are relatively similar to each other and regress to the average survivals, they are still significantly different (log-rank test p-value 8.60 × 10^−12^ and 9.75 × 10^−3^ for BRCA and GBM, respectively, Figs 3e and 4e). The correlations between all features and DEG patient groups are less obvious (Figs 3f and 4f), but two BRCA groups (1 and 3) preserve the patterns as having most patients as ER- and PR-, even though ER and PR status were excluded from DEG patient group identification. DEG patient group 3, the most comparable group to the original triple negative group (patient group 3), is also the group that has the worst survival curve (Fig. 3e). For GBM, DEG patient group 1, having a similar distribution in DEG modules, especially in DEG modules 1–5, as the original patient group 4, is also the one that has the best overall survival time (Fig. 4f). Comparisons of the DEG patient groups with known subtypes (PAM50 for BRCA and TCGA subtypes for GBM) were also carried out (Figs 5 and 6. Supplementary Tables S9 and S10). Even though the DEG patient groups were obtained without including any clinical feature that was involved in defining these subtypes, the correlation between DEG patient groups and subtypes still exists. For example, BRCA DEG patient groups 2, 3, and 4 have a single dominant PAM50 subtype, where group 3 is enriched in Basal subtype patients as expected (Fig. 5c,d). GBM DEG patient groups 2, 3, 4 and 6 have a single dominant TCGA subtype, where mesenchymal subtype is again divided into two subgroups (Fig. 6c,d). All these suggest that DEG modules alone are able to identify patient subgroups of distinct genetic aberration patterns with significantly different survival outcomes.

### Cox regression models

In order to evaluate the contribution of each feature towards survival estimation, we trained a Cox regression model using all features as covariates for all patients as a whole and for each patient group separately (Table 3). To compare clinical features and DEG modules, we also trained a Cox regression model using only clinical features and only DEG modules for all patients and for each DEG patient group (Supplementary Table S11). For BRCA, the all-patients model that received the highest concordance index (C-index) is the model trained using all covariates. Its C-index, 0.724, is higher than previously reported Cox regression models trained using only clinical and subtype information (0.67)^46^. For the patient-group-specific models, each patient group has a different combination of clinical features as significant (Wald-test p-value < 0.05). The DEG modules that are generally significant across all-patient and DEG patient groups are modules 1, 2 and 5. Modules 1 and 2 are positively correlated with the hazard rate, and module 5 is negatively correlated with the hazard rate. These partially explain the survival curves we observed above. With high value in module 2, patient group 4 has a much lower survival fraction compared to patient group 2, even though their other DEG modules are comparable. The lower average value in module 2 also resulted in a better survival outcome of DEG patient groups 2, 5 and 1. Note that the dominant SGAs of module 2 are *PTEN* and *PIK3CA*; a high value in this module represents activation of the PI3K/Akt signalling pathway that is known to be related to ILC^22^.

**Table 3:** The Cox regression models trained for BRCA and GBM for all patients and for each specific patient group, with different combinations of covariates. Significant covariates and C-index are listed for each model.

Cox regression model	BRCA	C-index	GBM	C-index
	Significant covariates (coefficient)		Significant covariates (coefficient)	
All patients-all covariates	ER status (−0.118)	0.724	age at diagnosis (0.486)	0.665
	Her2 status (0.122)		module 1 (−0.496)	
	age at diagnosis (0.196)		module 4 (0.374)	
	tumour histology type (−0.226)		module 11 (0.738)	
	lymph node assessment (0.264)			
	size of tumour (0.169)			
	module 2 (0.201)			
	module 5 (−0.205)			
Patient group 1-all covariates	tumour histology type (−0.504)	0.665	age at diagnosis (0.475)	0.684
	lymph node assessment (0.687)		module 7 (−0.479)	
	module 2 (0.450)		module 11 (1.816)	
Patient group 2-all covariates	Her2 status (0.335)	0.701	age at diagnosis (0.496)	0.688
	age at diagnosis (0.473)		module 2 (0.689)	
	lymph node assessment (0.179)		module 12 (1.400)	
	size of tumour (0.455)			
	module 1 (0.408)			
	module 2 (0.270)			
	module 5 (−0.497)			
Patient group 3-all covariates	tumour histology type (−0.605)	0.680	age at diagnosis (0.620)	0.624
	lymph node assessment (0.354)			
Patient group 4-all covariates	ER status (−0.508)	0.717	module 4 (1.079)	0.707
	PR status (−0.354)		module 8 (1.796)	
	age at diagnosis (0.311)		module 9 (−1.066)	
	lymph node assessment (0.368)			
	size of tumour (0.118)			
	module 2 (0.378)			
Patient group 5-all covariates	lymph node assessment (0.226)	0.680	age at diagnosis (0.384)	0.759
	size of tumour (0.248)		module 1 (−1.840)	
			module 2 (−1.144)	
			module 3 (1.862)	
			module 5 (−1.609)	
			module 6 (−1.711)	
			module 11 (2.743)	
			module 12 (1.343)	
Patient group 6-all covariates	NA		age at diagnosis (0.789)	0.720
			module 1 (−1.196)	
			module 5 (1.355)	
			module 11 (1.666)	

Unlike BRCA, where clinical features dominate survival estimation, most GBM Cox regression models contain several DEG modules as significant covariates. The most common significant DEG module across patient groups and DEG patient groups is module 11, with its dominant SGA *EGFR*. *EGFR* has been used as the primary marker in distinguishing between GBM patients and it was found to interact with multiple signalling pathways in GBM^47^. In addition to module 11, the set of significant DEG modules are mostly mutually exclusive across patient groups. In other words, even though GBM patients generally share similarly undesirable survival outcomes, their survival rates can be explained by different combinations of genetic features. This suggests that each of them took a different disease mechanism in their tumour developments. For example, module 7, the smallest DEG module with dominant SGA *EGFR* and *TP53*, has a high diversity across patients. This module represents the result of the communications between the Glioma pathways (KEGG map05214), which are known to explain the disease mechanism for both primary and secondary GBM^48^. In addition, the C-index of GBM Cox regression models is higher in the patient-group-specific model than in the overall model, which also supports the idea that different patient groups underwent different disease development procedures that should not be mixed. Three patient groups, 4, 5 and 6 (together containing 225 patients), have a C-index over 0.7, which is higher than a previously reported Cox regression model trained on a subset of TCGA GBM patients using clinical and imaging features (0.69)^49^.

## Discussion

In this study, we designed (and evaluated) a novel computational framework, which utilizes the causal inferences between SGAs and DEGs for constructing expression and signalling state representations, in the form of modules of DEGs that reflect the major transcriptomic programs that are perturbed in a cancer type. We conjecture that different combinations of expression status of DEG modules potentially reflect different combinations of aberrant pathways, or in other words, different disease mechanisms, which are informative towards clinical outcome predictions. Indeed, we have shown that different combinations of DEG modules divided BRCA and GBM patients into subgroups that exhibit significantly different survival patterns. Since the identification of DEG modules was driven by estimates of causal relationships between SGA and DEG events, our approach provides underlying mechanistic information for each cancer subtype, and such information can potentially be used to guide future targeted therapy in a pathway-oriented fashion. This differentiates our method from previous approaches of using gene expression data to discover cancer subtypes, which usually do not provide mechanistic information.

For identifying DEG modules from the networks, we chose to implement the spectral clustering algorithm. The major advantage of the spectral clustering algorithm is in its good performance in identifying modules with high data connectivity but not necessarily with high data compactness^50,51^. Specifically, since the DEG networks were constructed based on regulatory relationships between DEGs, we put more emphasis on identifying modules that connect sequences of DEGs rather than modules with a high direct correlation between any pair of DEGs. Such sequences of DEGs may represent cascades of aberrant signalling resulting from upstream perturbed genes. Two DEGs that are indirectly connected through a subsequence of other genes may still be controlled under the same regulatory network. In addition, our DEG networks were relatively dense (766,444 edges for BRCA, 1,567,144 edges for GBM), where classical hierarchical clustering or k-means would fail to untangle the correlations among DEGs and be unable to identify robust modules, no matter whether the correlations among DEGs were measured as co-regulation frequencies or more traditional expression profile distances (Supplementary Fig. S1 visualizes the consensus matrices of hierarchical clustering). Spectral clustering, as we showed here, would still be able to find stable and consistent modules across different independent random initializations.

For general clustering or communication detection algorithms, features with the highest diversity across data will be given a higher priority to be used to cut between observations, which maximizes both the distance between observations of different resulting clusters and similarity between observations in the same cluster. For gene expression data, genes that are tissue-specific are often more diverse across samples than other globally expressed genes. Consequently, using solely gene expression data or genetic signatures like PAM50 for discovering cancer subtypes often leads to a division of subtypes based on cell-of-origin. The approach we used to identify patient groups with a combination of clinical features and DEG modules, however, does not suffer from this problem. For example, none of the BRCA patient groups or DEG patient groups is overwhelmingly dominated by a single PAM50 subtype that related to a cell type. The division of ILC and IDC + ILC in patient groups 1, 2 and 4 also supports that our patient groups are not simply tissue-specific divisions. In addition, each patient group presents a distinct pattern of DEG modules, where each module reflects the compositive effect of a group of genes and provides information about the status of signalling pathway perturbations that drives tumorigenesis. All these suggest that our approach is robust to tissue-specific-expressions and can identify subtypes that are disease mechanism indicative. In the meantime, the patient groups present distinct survival outcomes, which are crucial for being used as a clinical guidance tool. Specifically, we are expecting that our BRCA patient groups can serve as an alternative for the PAM50 subtypes.

In general, clinical features seem to be more informative about survival than DEG modules in BRCA. One of the reasons is that certain clinical features are indeed molecular features, including the ER, Her2 and PR status, which are not independent from the DEG modules. For example, the Her2 expression status measured using immune histology is correlated with the expression status of the DEG module driven by dominant SGAs *ERBB2* and *TP53*. As a result, the corresponding DEG modules became less significant in Cox regression due to the redundant information. The decrease in C-index when DEG modules were excluded (Supplementary Table S11), and the irreplaceable role that DEG modules play in GBM survival estimation, support that these DEG module features preserved independent pathway-oriented information that clinical features did not capture.

## Methods

### Significant TCI causal inference generation

The TCI algorithm is a Bayesian Causal Network model, which models the SGAs and the DEGs as a bipartite graph and adds edges between the two gene sets that represent causal relationships^16^. In particular, for each tumour sample, the algorithm assigns each DEG one, and only one, SGA as its cause by comparing all candidate SGAs based on the BDeu scoring; each SGA can be assigned to multiple DEGs (Fig. 1). The biological intuition behind this is that the differential expression of a gene is mainly due to the direct interaction between this gene and a single SGA; all indirect interactions between the gene and other SGAs are relatively trivial if the direct interaction is recognized. On the other hand, one SGA can affect the expression status of multiple genes at the same time. In the TCI algorithm, a gene is considered a somatic alteration carrier if one or more somatic mutations (SM), or somatic DNA copy number alterations (SCNA), were observed on it; a gene is recognized as a DEG if its expression level significantly deviates from the mean of its expression distribution in healthy tissue. The TCI causal inferences we used were produced by running TCI with a combination of SM, SCNA (for identifying SGAs), and expression (for identifying DEGs) data of 5,097 tumours across 16 cancer types (includes 891 BRCA tumours and 144 GBM tumours)^16^. The inferences were further filtered through a series of empirical standards to obtain robust and significant results. The filtering standards we used are:

- A SGA-DEG causal relationship is considered valid if its posterior probability is larger than the posterior probability estimated in a random permutated experiment.
- A SGA is called a driver in a tumour if TCI assigns it to be a cause of 5 or more DEGs in the tumour.
- A SGA is called a significant driver if it is called driver in 30 or more tumours AND it is called driver in at least 25% of tumours where it is observed as a SGA.
- A SGA-DEG is called a significant causal relationship if the SGA is a significant driver AND the DEG is caused by this SGA in at least 50 tumours OR 20% of the tumours where the SGA is called a driver.

Some tumour samples contain no significant inference after filtering. Consequently, the significant inferences we used for BRCA and GBM analyses were from 874 BRCA tumour samples and 143 GBM tumour samples, respectively. For a more detailed overview of the data generation and processing procedure, please refer to the original TCI paper^16^.

### DEG module identification

#### DEG network construction

The TCI significant inferences were used to construct DEG networks in the form of a weighted, undirected graph. When constructing the graph for a single cancer type, the corresponding subsets of significant inferences were extracted. Each node in this graph represents a DEG that was identified in more than 10% of the tumours. Edges were added between DEG pairs where the two DEGs were co-regulated in the same tumour by the same SGA. The edge weight is defined as the frequency of the co-regulation, which equals the number of tumours in which the co-regulation took place. The weighted, undirected graph was represented in the form of a symmetric affinity matrix, where the affinity in row *i* column *j* is the edge weight between DEG*i* and DEG*j*.

#### Spectral clustering

The spectral clustering we implemented to identify modules from the DEG networks was derived from the algorithm described by Ng, 2002^18^. In our implementation, a DEG network affinity matrix is first converted to a pseudo-distance matrix by taking the inverse of each affinity value. It is then transformed into an optimized affinity matrix with the Gaussian kernel, as shown in Equation (1)

1 $$\begin{array}{c}{A}_{ij}=\exp ({D}_{ij}^{2}/2{\sigma }^{2})\end{array}$$

Here *D*_*ij*_ and *A*_*ij*_ are the pseudo-distance and optimized affinity between *DEG*_*i*_ and *DEG*_*j*_. The standard deviation *σ* of the Gaussian kernel is selected based on the distribution of pseudo-distances to convert short distances to high affinities and suppress long distances (0.05 for BRCA and 0.1 for GBM). The remaining steps are identical to steps 2–6 in the standard spectral clustering algorithm^18^. In particular, a Laplacian matrix is computed from the optimized affinity matrix, from which the *k* largest eigenvectors are extracted to project the data into a *k* dimensional feature space. The data points are then clustered via the k-means algorithm.

With the use of k-means, the clustering result partially depends on the random initialization of the *k*-centres of clusters. To determine the value of *k* (i.e., the number of DEG modules), we first tried consensus spectral clustering with *k = *5, 10, 15, 20, then narrowed down to the range between the two adjacent *k*s that gave the most stable consensus matrices, and tried each *k* from the range. For generating the consensus matrix of each *k*, a spectral clustering was repeated independently for 100 times with different random initializations. The value of *k* was selected such that further increasing *k* would result in modules that were unstable, with significant overlaps across modules on the consensus matrix. Such overlaps indicated that the data points that were finally assigned to two different modules were often clustered into the same group across independent runs. This suggests that the two modules should be merged and the *k* being used was too large. The module assignments used in constructing the survival features were generated by running the clustering algorithm one more time with the selected *k*.

### Survival analysis

#### Dataset construction

In constructing the dataset for survival analyses, each DEG module identified by spectral clustering was treated as a single feature and was represented with the mean of the expression levels of all DEGs in the module. This representation can be seen as a surrogate measure for the aberration status of the signalling pathway that each module represents. Other clinical features of interest (e.g. age at diagnosis, etc.) were also added. For BRCA, the gene expression, clinical and survival data used were from the METABRIC project^20^, accessed through the Synapse repository (synapse.sagebase.org, ID syn1688369). The experimental protocol for the METABRIC data has been approved by the University of Pittsburgh Institutional Review Board (IRB# PRO18010238). For GBM, the microarray gene expression data and clinical data were downloaded from TCGA through the Firehose browser of the Broad Institute. All computational methods applied on the data in this study were carried out in accordance with relevant guidelines and regulations of the METABRIC dataset and TCGA database. The DEG modules were obtained with TCI inferences that were produced using RNA-seq data from the TCGA database, and some DEGs were not available from the METABRIC expression data or the TCGA GBM microarray data. As a result, the number of DEGs used to compute each DEG module feature was smaller than the original number of DEGs in each module. We refer to these DEGs as the effective DEGs (Tables 1 and 2). From the METABRIC clinical data, we extracted eight features and added these to our BRCA dataset–the age at diagnosis, size of tumour, grade of disease, lymph node assessment, tumour histology type, ER status, PR status, and Her2 status. For tumour histology type we only considered three factor levels, including IDC-TUB, IDC-MUC, and IDC-MED. For GBM, patient age at diagnosis was extracted and added to our dataset as the only clinical feature. Since clinical features typically took different scales of values, all features (including DEG module features) were normalized across patients by subtracting the mean of values and dividing by the standard deviation. The final survival dataset took the form of a table in which each patient was represented with a feature vector, a survival time, and a binary value indicating the death status (0 for alive and 1 for dead). The BRCA survival feature dataset contained 1,981 patients and the GBM dataset contained 524 patients.

#### Patient groups identification

Patient groups were identified using consensus clustering, with PAM clustering as the base method. The consensus clustering function was from the R package *ConsensusClusterPlus*^52^, version 1.38.0. The number of patient groups was determined using the consensus matrix and the area under the consensus cumulative distribution function curve (AUCDFC). This was done by clustering with the number of groups that varies from 2 to 15 (200 resamplings for GBM, 100 resamplings for BRCA) and selecting the point at which there was no significant overlap between the resulting groups on the consensus matrix and at which further increasing the number of groups would not lead to a significant increase in the AUCDFC.

#### Patient groups survival models

The Kaplan-Meier plot of each patient group was generated using the R package *survival*^53^ version 2.41.3. The same package also provided the functions we used for doing the log-rank test and the Cox regressions. The prediction performances of various Cox regression models were compared by computing the C-index of the model on the survival data^54,55^.

### Figure processing

All multipart figures were prepared using Adobe Photoshop CS6 version 13.0. Contrast was adjusted for Figs 2 and 3, and the adjustment was applied equally across both entire figures.

## Supplementary information

Supplementary Figure

Supplementary Table S1-S11

## Data Availability

The TCI dataset generated and analysed during this study is available from the corresponding author upon request. The source code for spectral clustering is freely available for download at https://github.com/evasnow1992/SpectralClustering.
