# Supplementary material for: Tumour-specific Causal Inference Discovers Distinct Disease Mechanisms Underlying Cancer Subtypes
Source: Sci Rep. 2019 Sep 13;9:13225. doi: 10.1038/s41598-019-48318-7 (PMC6744493; doi:10.1038/s41598-019-48318-7)
Supplement: Supplementary file 1 — Supplementary Figure [file 41598_2019_48318_MOESM1_ESM.pdf]

Tumour-specific Causal Inference Discovers Distinct Disease Mechanisms Underlying Cancer Subtypes

Yifan Xue[1], Gregory Cooper[1], Chunhui Cai[1], Songjian Lu[1], Baoli Hu[2,3,4], Xiaojun Ma[1], Xinghua Lu[1,*]

[1]Department of Biomedical Informatics, University of Pittsburgh School of Medicine, Pittsburgh, 15260, United States
[2]Department of Neurological Surgery, University of Pittsburgh School of Medicine, Pittsburgh, 15260, United States
[3]Paediatric Neurosurgery, UPMC Children's Hospital of Pittsburgh, Pittsburgh, 15213, United States
[4]Molecular and Cellular Cancer Biology Program, UPMC Hillman Cancer Centre, Pittsburgh, 15232, United States
[*]xinghua@pitt.edu

Supplementary figure file.

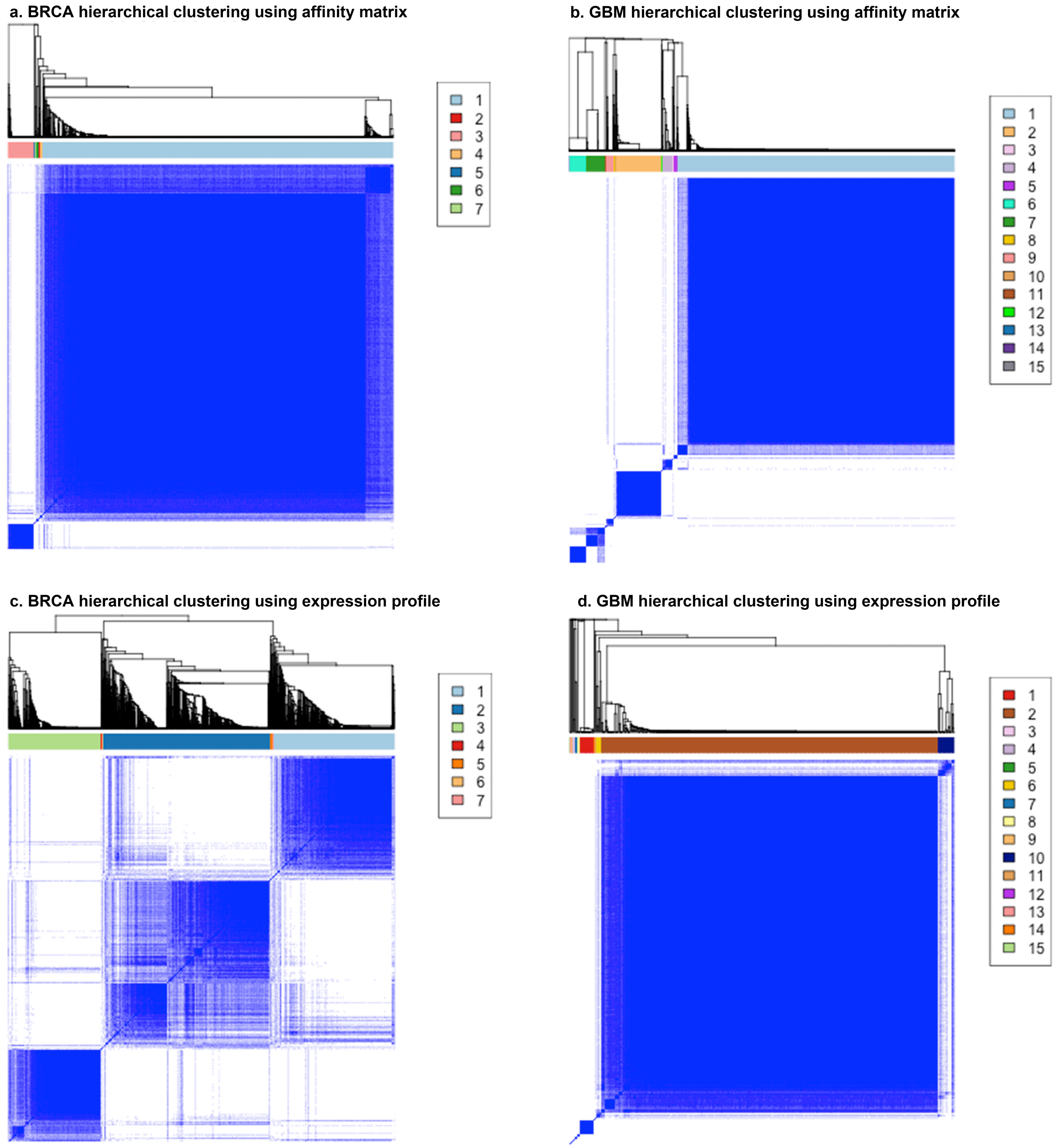

Figure S1. The consensus matrices of different clustering methods for identifying DEG modules, including hierarchical clustering using affinity matrix (a and b), and hierarchical clustering using expression profile with *1 - Pearson correlation* as distance (c and d). The higher the frequency two DEGs are clustered into the same module, the darker blue the corresponding spot on the matrix. Each block sitting on the diagonal corresponds to a DEG module. The single dominant module composed of the majority of genes in a, b and d, and the overlapping across modules in c suggest that hierarchical clustering was unable to untangle the correlations between DEGs to identify robust modules, no matter whether the correlations were measured as the co-regulation frequency (affinity) or expression profile distance.
